# Role of the Proteasome in Excitotoxicity-Induced Cleavage of Glutamic Acid Decarboxylase in Cultured Hippocampal Neurons

**DOI:** 10.1371/journal.pone.0010139

**Published:** 2010-04-12

**Authors:** Márcio S. Baptista, Carlos V. Melo, Mário Armelão, Dennis Herrmann, Diogo O. Pimentel, Graciano Leal, Margarida V. Caldeira, Ben A. Bahr, Mário Bengtson, Ramiro D. Almeida, Carlos B. Duarte

**Affiliations:** 1 Center for Neuroscience and Cell Biology, Department of Life Sciences, University of Coimbra, Coimbra, Portugal; 2 Biotechnology Research and Training Center, University of North Carolina, Pembroke, North Carolina, United States of America; 3 Department of Cancer and Cell Biology, Genomics Institute of the Novartis Research Foundation (GNF), San Diego, California, United States of America; Universidade Federal do Rio de Janeiro (UFRJ), Brazil

## Abstract

Glutamic acid decarboxylase is responsible for synthesizing GABA, the major inhibitory neurotransmitter, and exists in two isoforms—GAD65 and GAD67. The enzyme is cleaved under excitotoxic conditions, but the mechanisms involved and the functional consequences are not fully elucidated. We found that excitotoxic stimulation of cultured hippocampal neurons with glutamate leads to a time-dependent cleavage of GAD65 and GAD67 in the N-terminal region of the proteins, and decrease the corresponding mRNAs. The cleavage of GAD67 was sensitive to the proteasome inhibitors MG132, YU102 and lactacystin, and was also abrogated by the E1 ubiquitin ligase inhibitor UBEI-41. In contrast, MG132 and UBEI-41 were the only inhibitors tested that showed an effect on GAD65 cleavage. Excitotoxic stimulation with glutamate also increased the amount of GAD captured in experiments where ubiquitinated proteins and their binding partners were isolated. However, no evidences were found for direct GADs ubiquitination in cultured hippocampal neurons, and recombinant GAD65 was not cleaved by purified 20S or 26S proteasome preparations. Since calpains, a group of calcium activated proteases, play a key role in GAD65/67 cleavage under excitotoxic conditions the results suggest that GADs are cleaved after ubiquitination and degradation of an unknown binding partner by the proteasome. The characteristic punctate distribution of GAD65 along neurites of differentiated cultured hippocampal neurons was significantly reduced after excitotoxic injury, and the total GAD activity measured in extracts from the cerebellum or cerebral cortex at 24h postmortem (when there is a partial cleavage of GADs) was also decreased. The results show a role of the UPS in the cleavage of GAD65/67 and point out the deregulation of GADs under excitotoxic conditions, which is likely to affect GABAergic neurotransmission. This is the first time that the UPS has been implicated in the events triggered during excitotoxicity and the first molecular target of the UPS affected in this cell death process.

## Introduction

In traumatic brain injury, epilepsy, and following episodes of hypoxia-ischemia the excessive release of glutamate and the consequent overactivation of glutamate receptors leads to cell death by excitotoxicity [1]–[4]. Brain ischemia also has a strong impact in GABAergic neurotransmission. The Ca^2+^-dependent exocytotic release of GABA appears to account for the initial phase of neurotransmitter release at the onset of ischemia, while the reversal of the plasma membrane transporters is responsible for much of the subsequent efflux [5], [6]. However, the decrease in surface expression of post-synaptic GABA_A_ receptors, in part due to their internalization, decreases GABAergic synaptic transmission [7]. Following transient focal ischemia there is also a decrease in the expression of the vesicular GABA transporter, which may have a delayed impact on the exocytotic release of the neurotransmitter [8]. The plasma membrane GABA transporter GAT1 is a calpain substrate [9], and calpain activation in the postischemic brain [10] may contribute to the deregulation of the transporter.

Glutamic acid decarboxylase (GAD) is the key enzyme in the synthesis of γ-aminobutyric acid (GABA) [11] and any alterations in the activity of the enzyme will also have an impact on the GABAergic synaptic transmission. GAD exists in two isoforms encoded by different genes, GAD65 and GAD67, with a molecular weight of 65 and 67 KDa, respectively [12]. GAD65 represents 81% of total GAD in rat hippocampus [13], and is found predominantly in association with synaptic vesicle membranes in nerve terminals [14]–[16]. This GAD isoform synthesizes mainly the vesicular pool of GABA [17], [18], and is responsible for the fine tuning of inhibitory transmission [19]. In contrast, GAD67 is evenly distributed throughout the cell [20], being constitutively active and accounting for the basal production of the cytosolic pool of GABA [21]. Both isoforms of GAD are cleaved in cerebrocortical neurons subjected to excitotoxic conditions by a mechanism that is sensitive to inhibitors of calpain [22]–[25], a non-lysosomal, calcium-activated protease that has been implicated in excitotoxic neuronal damage [26], and recombinant GAD65 and 67 are cleaved in vitro by calpain [22], [23]. Cathepsin inhibitors also inhibited the cleavage of GAD65 and 67 in cerebrocortical neurons exposed to a toxic concentration of glutamate, and recombinant GAD was cleaved by cathepsin L in an in vitro assay [24]. These evidences suggest that multiple proteolytic systems are involved in the cleavage of GAD under excitotoxic conditions.

The ubiquitin-proteasome system (UPS) is the major extralysosomal system for protein degradation in the cells [27], [28]. Proteins targeted to be degraded by this system are first conjugated by polyubiquitin chains and then degraded by the proteasomes. The role of the UPS in cell death in the ischemic brain is rather complex since the activity of the proteasome is downregulated in the ischemic brain [29], [30] but inhibition of the proteasome was found to be neuroprotective in focal brain ischemia [31]–[33]. Furthermore, the effect of proteasome deregulation on the turnover of specific proteins in the ischemic brain remains to be investigated. Hence, in the present study we investigated the putative role of the UPS in GAD cleavage under excitotoxic conditions. In particular the soluble isoform GAD67 is present mainly in the cytoplasm [34] and, therefore, may constitute a target of the UPS. We found that excitotoxic stimulation of hippocampal neurons with glutamate downregulates GADs, both at mRNA and protein levels. Our results indicate that the UPS does regulate GAD67 cleavage under excitotoxic conditions, possibly through modulation of an unknown GAD binding partner. Cleavage of GADs diminished the activity of the enzyme and the characteristic punctate distribution of GAD65 along neurites was also affected under excitotoxic conditions.

## Materials and Methods

### Hippocampal cultures

Primary cultures of rat hippocampal neurons were prepared from the hippocampi of E18-E19 Wistar rat embryos, after treatment with trypsin (0.06%, for 15min at 37°C; GIBCO-Invitrogen, Paisley, UK) and deoxyribonuclease I (5.36 mg/ml), in Ca^2+^- and Mg^2+^-free Hank's balanced salt solution (HBSS; 5.36 mM KCl, 0.44 mM KH_2_PO_4_, 137 mM NaCl, 4.16 mM NaHCO_3_, 0.34 mM Na_2_HPO_4_.2H_2_O, 5 mM glucose, 1 mM sodium pyruvate, 10 mM HEPES and 0.001% phenol red). The hippocampi were then washed with HBSS containing 10% fetal bovine serum (GIBCO-Invitrogen), to stop trypsin activity, and transferred to Neurobasal medium (GIBCO-Invitrogen) supplemented with B27 supplement (1∶50 dilution; GIBCO-Invitrogen), 25 µM glutamate, 0.5 mM glutamine and 0.12 mg/ml gentamycin. The cells were dissociated in this solution and were then plated in 6 well plates (870,000 cells/well) coated with poly-D-lysine (0.1 mg/mL), or on poly-D-lysine coated glass coverslips, at a density of 150,000 cells/well (12 well plates). The cultures were maintained in a humidified incubator of 5% CO_2_/95% air, at 37°C, for 7 or 10 days. Excitotoxic stimulation was performed with 125 µM glutamate in supplemented Neurobasal medium, for 20 min at 37°C, in a humidified incubator. After stimulation with glutamate the cells were further incubated with the original culture medium for the indicated periods of time. When appropriate, 50 µM UBEI-41 (ubiquitin-activating enzyme inhibitor; Biogenova Corp., Maryland, USA), 1 µM MG132 (Calbiochem, Darmstadt, Germany), 10 µM Lactacystin (Sigma, MO, USA) or 10 µM YU102 (Biomol, Exeter, UK) were added to the incubation medium 30 min (or 1 h for UBEI-41) before stimulation.

Animals used in the preparation of cell cultures and in the GAD activity experiments (see below) were handled according to National and Institutional guidelines. Experiments conducted at the Center for Neuroscience and Cell Biology were performed according to the European Union Directive 86/609/EEC on the protection of animals used for experimental and other scientific purposes. These experiments did not require approval by an Institutional Animal Care and Use Committee (IACUC). The work performed at GNF adhered to the Animal Behavior Society Guidelines for the Use of Animals in Research, and was approved by the Institutional Animal Care IACUC.

### Preparation of extracts

Hippocampal neurons (DIV7) were washed twice with ice-cold PBS and once more with PBS buffer supplemented with 1 mM DTT and a cocktail of protease inhibitors (0.1 mM PMSF; CLAP: 1 µg/ml chymostatin, 1 µg/ml leupeptin, 1 µg/ml antipain, 1 µg/ml pepstatin; Sigma-Aldrich Química, Sintra, Portugal). The cells were then lysed with RIPA (150 mM NaCl, 50 mM Tris-HCl, 5 mM EGTA, 1% Triton, 0.5% DOC and 0.1% SDS at a final pH 7.5) supplemented with the cocktail of protease inhibitors. After centrifugation at 16,100 g for 10 min, protein in the supernatants was quantified using the bicinchoninic acid (BCA) assay (Thermo Scientific, Rockford, IL), and the samples were denaturated with 2x concentrated denaturating buffer (125 mM Tris, pH 6.8, 100 mM glycine, 4% SDS, 200 mM DTT, 40% glycerol, 3 mM sodium orthovanadate, and 0.01% bromophenol blue), at 95°C for 5 min.

### Total RNA isolation

Total RNA was extracted from 7 DIV cultured hippocampal neurons using TRIzol® Reagent (Invitrogen), following the manufacturer's specifications. The content of 2 wells from a 6 well plate, with 870,000 cells/well (DIV7), was collected for each experimental condition. After the addition of chloroform and phase separation, the RNA was precipitated by the addition of isopropanol. The precipitated RNA was washed once with 75% ethanol, centrifuged, air-dried and resuspended in 60 µl of RNase-free water (GIBCO-Invitrogen). The whole procedure was performed at 4°C.

### RNA Quality and RNA Concentration

RNA quality and integrity was assessed using the Experion automated gel-electrophoresis system (Bio-Rad, Amadora, Portugal), as previously described [35]. A virtual gel was created for each sample, allowing the detection of degradation of the reference markers, RNA 18S and 28S. Samples showing RNA degradation or contamination by DNA were discarded. RNA concentration was determined using both the fluorescent dye RiboGreen (Invitrogen-Molecular Probes, Leiden, The Netherlands) and NanoDrop 1000 (Thermo Scientific). The samples were aliquoted and stored at -80°C to further use.

### Reverse Transcription reaction

For first strand cDNA synthesis 1000 ng of total RNA was mixed with Random Hexamer Primer p(dN)_6_ followed by 10 min denaturation at 65°C to ensure loss of secondary structures that may interfere with the annealing step. The samples were chilled on ice, and the template-primer mix was then supplemented with Reaction Buffer (50 mM Tris/HCl, 30 mM KCl, 8 mM MgCl_2_, pH 8.5), Protector RNase Inhibitor (20U), dNTPs (1 mM each) and finally AMV Reverse Transcriptase (10U; Roche, Carnaxide, Portugal), in a 20 µl final volume. The reaction was performed at 25°C for 10 min, followed by 30 min at 55°C, for primer annealing to the template and cDNA synthesis, respectively. The Reverse Transcriptase was then denatured during 5 min at 85°C, and the samples were then cooled to 4°C for 5 min, and finally stored at −80°C until further use.

### Primer Design

Primers for real-time PCR were designed using the “Beacon Designer 7” software (Premier Biosoft International, CA, USA), and the following considerations were taken: (1) GC content about 50%; (2) annealing temperature (T_a_) between 55±5°C; (3) secondary structures and primer-dimers were avoided; (4) Primer length between 18–24 bp; (5) Final product length between 100–200 bp. The primers used for amplification of GAD65 and GAD67 were, respectively, NM 012563 (accession number to mRNA sequence) – 5′GCT CAT TGC CCG CTA TAA G3′ and 5′ATC ACG CTG TCT GTT CCG3′; NM 017007 – 5′ACA CTT GAA CAG TAG AGA C3′ and 5′GCA GGT TGG TAG TAT TAG G3′. The primers used for the amplification of endogenous controls GAPDH and Tubulin alpha 1a were, respectively, NM 017008 –5′AAC CTG CCA AGT ATG ATG3′ and 5′ GGA GTT GCT GTT GAA GTC3′ ; NM 022298 –5′CAT CCT CAC CAC CCA CAC3′ and 5′GGA AGC AGT GAT GGA AGA C3′. Following the first experiment all sets of primers were tested for their specificity in an agarose gel that allows determination of the product size and possible non-specific products.

### Real-Time PCR

For gene expression analysis 2 µl of 1∶100 diluted cDNA was added to 10 µl 2x SYBR Green Master Mix (Bio-Rad) and the final concentration of each primer was 250 M in 20 µl total volume. The thermocycling reaction was initiated with activation of the Taq DNA Polymerase by heating at 95°C during 30 s, followed by 45 cycles of a 10 s denaturation step at 95°C, a 30 s annealing step, and a 30 s elongation step at 72°C. The fluorescence was measured after the extension step, using the iQ5 Multicolor Real-Time PCR Detection System (Bio-Rad). After the thermocycling reaction the melting step was performed with slow heating, starting at 55°C and with a rate of 0.5°C per 10 s, up to 95°C, with continuous measurement of fluorescence, allowing detection of possible non-specific products. The assay included a non-template control and a standard curve (in 10-fold steps) of cDNA for assessing the efficiency of each set of primers. All reactions were run in duplicate to reduce confounding variance [36].

### Real Time PCR Data Processing

The threshold cycle (C_t_) represents the detectable fluorescence signal above background resulting from the accumulation of amplified product, and is a proportional measure of the starting target sequence concentration. C_t_ was measured in the exponential phase and, therefore, was not affected by possible limiting components in the reaction. For every run performed C_t_ was set at the same fluorescence value. Data analysis was performed using the GenEx (MultiD Analyses, Sweden) software for Real-Time PCR expression profiling, and the results were normalized with a set of two internal control genes. Statistical analysis was performed using the Student's *t* test.

### Immunoblotting

Protein samples were separated by SDS-PAGE, in 12% polyacrylamide gels (or 7.5% gels when spectrin products were detected), transferred to polyvinylidene (PVDF) membranes (Millipore Corp., Billerica, MA), and immunoblotted. Blots were incubated with primary antibodies (overnight at 4°C), washed and exposed to alkaline phosphatase-conjugated secondary antibodies (1∶20000 dilution; 1 h at room temperature) or exposed directly to ECL in the ubiquitin-conjugates detection which films were scanned and the optical densities of the bands were measured with appropriate software. Alkaline phosphatase activity was visualized by ECF on the Storm 860 Gel and Blot Imaging System (GE Healthcare, Buckinghamshire, UK). The following primary antibodies were used: anti-GAD65/67 (1∶5000, Sigma), anti-GAD67 (1∶250; BD Biosciences, Erembodegem, Belgium), antibody against calpain-mediated fragment of spectrin/fodrin nSBDP _N_SBDPs [1∶300 [37], [38]] and anti-β-Actin (1∶5000, Sigma).

### Recombinant GAD65 cleavage assay

0.75 µg of recombinant GAD65 (Diamyd Diagnostics, Stockholm, Sweden) were incubated with 1.5 µg of 20S or 26S proteasome (Biomol) at 37°C for 2 h, in a total volume of 20 µl of buffer (30 mM TrisHCl pH 7.6, 100 mM NaCl, 1 mM CaCl_2_, 2 mM MgCl_2_, 50 mM ATP, 1 mM DTT, 5% (v/v) glycerol), with or without 10 µM MG132. A pre-incubation of 5 min with the proteasome inhibitor was performed. Reactions were stopped by addition of 20 µl of 2x concentrated denaturating buffer (same for immunoblot), resolved by 12% PAGE and probed with a GAD65 antibody by Western blot.

### Purified proteasome activity

To test for the activity of the purified proteasome activity, the 20S and 26S proteasome preparations were incubated in the presence of the chymotrypsin-like fluorogenic peptide suc-LLVY-MCA (Peptide Institute, Inc., Osaka, Japan). The proteasome preparations were incubated with the substrate (50 µM) in the presence or in the absence of the proteasome inhibitor MG132 (10 µM), in a medium containing 1 mM EDTA, 10 mM tris-HCl (pH 7.5), 20% glycerol, 4 mM DTT, 2 mM ATP (100 µl final volume). Substrate degradation by the proteasome was monitored every 5 min during 1 h at 37°C in a fluorescence-luminescence detector (Synergy™ HT Multi-Mode Microplate Reader, BioTek, Winooski, VT), set to 380 and 460 nm, excitatory and emission wavelengths, respectively.

### Immunoprecipitation Assay

Immunoprecipitation of ubiquitin-conjugated proteins was performed using the Ubiqapture – Q Kit (Biomol, Exeter, UK), as described by the manufacturer. A total of 50 µg lysates from cultured hippocampal neurons were used per assay. Samples were added to the tubes containing 80 µl UbiQapture-Q matrix and incubated overnight at 4°C in an horizontal rotor mixer. The matrix was then carefully washed and the ubiquitin-protein conjugates were eluted by addition of 160 µl of PBS and 50 µl of 5X concentrated denaturating buffer (same for immunoblot). Samples were quenched by incubation during 15 min at 4°C on an horizontal rotor and then denaturated by heating during at 95°C for 10 min. The eluted fraction was clarified from the matrix pellet by centrifugation at 16,100 g during 10 min. Western blot analysis was performed as previously described using an anti-GAD65/67 antibody and the ubiquitin-conjugate antibody supplied by the kit, applying equal sample volumes (approximately 60 µl).

### Measurement of GAD activity

Wistar adult rats were decapitated and each head was covered and kept at room temperature (approximately 21°C) for 24 h. Brains were then dissected and placed on an ice-cold plate for dissection of the cerebellum and cerebral cortex. Samples were then resuspended in 50 mM TrisHCl and 0.02% Triton X-100, sonicated with a probe sonicator in 5 pulses of 5 seconds, and centrifuged at 16,100 g for 10 min. The supernatants were diluted (1∶30–1∶100) and the protein content was measured using the BCA method. Activity of glutamate decarboxylase (GAD) was measured by the [^14^C]CO2 trapping method, using L-[1-^14^C]-glutamic acid (60 mCi/mmol, GE Healthcare, Buckinghamshire, UK) as a substrate [39]. Enzyme activity was expressed as nmol of product/h/mg of protein. Reactions contained 40 µg of extract protein and 0.5 M KH_2_PO_4_, 5 mM ethylenediamineteraacetic acid (EDTA), 1 mM 2-aminoethyliso-thiouronium bromide (AET), 10 mM glutamate, 1 mM pyridoxal phosphate and L-[1-^14^C]-glutamic acid in a total volume of 100 µl. Samples were incubated for 1 h at 37°C in test tubes containing #32 glass fiber filters (Schleider and Schuell, Keene, NH, USA) coated with 0.5 M Solvable (Packard Instruments, CT, USA). Each filter was suspended at the top of the tube, just underneath a rubber stopper, which sealed the tube. The reaction was stopped by the injection of 15% trichloroacetic acid through the stopper. The tubes were incubated at room temperature for another 120 min to ensure complete release and absorption of [^14^C]CO_2_ into the filter paper. The filter papers were then removed from the tubes and placed in scintillation vials for measurement of the [^14^C]CO_2_ product in a Packard 2000 spectrometer provided with dpm correction. The scintillation cocktail used contained 5.84 g 2,5-diphenyloxazole (PPO) and 133.6 mg of 1,4-Bis(5-phenyl-2-oxazolyl)benzene (POPOP), 800 ml toluene and 200 ml of Triton X-100. Sample extracts were also analysed by Western Blot using the anti-GAD65/67 antibody.

### Immunocytochemistry

**Figure 1:**
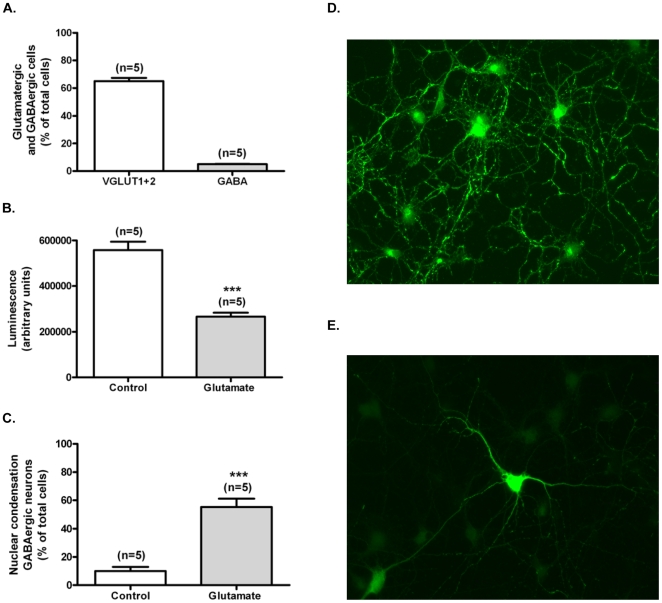
Glutamate excitotoxicity decreases viability of cultured hippocampal GABAergic neurons. GABAergic and glutamatergic neurons in the cultures (DIV7) were identified by immunocytochemistry, using antibodies against GABA (A, E) and VGLUT1+2 (A, D). The total number of cells present in the analysed fields was calculated based on the number of nuclei, stained with the fluorescent dye Hoechst 33342. Data are presented as mean±SEM of 5 independent preparations (A). Excitotoxic stimulation of hippocampal neurons was performed by incubation with 125 µM glutamate, for 20 min, in fresh Neurobasal medium containing B27 supplement, and the cells were further incubated in the original medium for 14 h. Cell death was assessed with the recombinant Luciferase chemoluminescence assay with CellTiterGlo (B), or by fluorescence microscopy using the fluorescence dye Hoechst 33342 (C). In the latter condition GABAergic cells were identified by immunocytochemistry, using an antibody against GABA. Data are presented as mean±SEM of 5 independent experiments. Statistical analysis was performed using Student's *t*-test. ***p<0.001.

For immunocytochemistry, cultured hippocampal neurons were grown on poly-D-lysine coated glass coverslips, at a density of 45×10^3^ cells/cm^2^, and were then fixed in PBS supplemented with 4% paraformaldehyde/4% sucrose, for 30 min at 4°C. After fixation the cells were permeabilized with 0.25% Triton X-100 in PBS, for 5 min at room temperature, washed three times in PBS, and then blocked with 20% normal goat serum, for 1 h at room temperature, and stained against VGLUT1 (1∶1000; Synaptic Systems) + VGLUT2 (1∶500; Synaptic Systems) or GABA (1∶2000; SIGMA) overnight at 4°C. Next, the cells were washed six times and incubated for 1 h at room temperature with the secondary antibody (Alexa Fluor® 488 goat anti-rabbit, 1∶500 to 1∶1000; Barcelona, Spain). The cells were washed three times, mounted on glass slides with the Dako mounting medium and viewed on an Axiovert 200 fluorescence microscope coupled to an Axiocam HRm digital camera (Zeiss) (Figure 1).

Immunocytochemistry experiments for localization of GAD65 were performed using hippocampal neurons maintained in culture for 10 days. The cells were fixed with 4% formaldehyde, 4% sucrose in PBS for 12 min at room temperature and were subsequently permeabilized with 0.25% Triton X-100 in PBS for 3 min, washed 3 times in PBS and incubated in blocking solution (2% bovine serum albumin, 2% glycine, and 0.2% gelatin in 50 mM NH_4_Cl) for 1 h at room temperature. Afterwards, the neurons were incubated for 1 h with a mouse monoclonal antibody anti-GABA_A_Receptor β2/3 (Upstate Biotechnology) at a dilution of 1∶200, and a rabbit polyclonal antibody anti-GAD65 (SIGMA) at a dilution of 1∶1000, in blocking solution. Following the incubation with the primary antibodies, the cells were rinsed 4 times in PBS during 15 min periods, incubated for 1 h at room temperature with secondary antibodies Alexa Fluor 568 goat anti-rabbit and Alexa Fluor 488 goat anti-mouse (Invitrogen), at a dilution of 1∶500 in blocking solution, rinsed 3 times in PBS followed by a final rinse in deionized water, dried, and mounted in Vectashield mounting solution (Vector Laboratories, Inc.). The neurons were imaged at the Garner Laboratory (Stanford University) with a Zeiss Axovert 200M microscope using a 63x objective. The image emission was directed through a CSU10 spinning disk confocal unit (Yokogawa) and collected by a 512B-CCD camera (Roper Scientific). Image acquisition and analysis was conducted with Metamorph software. For each field of view, stacks of 2 images with a Z step size of 0.2 mm were collected and a Metamorph 3D reconstruction tool was used to create a projection image. For each condition, 15 images were collected from two different cover slips.

For quantitative assessment of GABA_A_Receptor β2/3 and GAD65 protein co-localization, the 3D reconstruction stacked images were submitted to threshold using the MetaMorph Inclusive Threshold application, in order to include only the puncta labeled by the GAD65 antibody. The total number of GAD65 puncta was determined using Integrated Morphometry Analysis of the image and selecting the Area parameter setup for measurement. After proceeding to perform an identical 3D reconstruction and threshold of the GABA_A_Receptor β2/3 corresponding image, the two reconstructed images were overlayed and a color threshold was set. Finally, the overlay image was used to quantify the number of puncta positive for both GABA_A_Receptor β2/3 and GAD65, using the Integrated Morphometry Analysis tool. This procedure was repeated for each field of view, and the ratio of GABA_A_Receptor β2/3 subunits and GAD65 positive puncta per total number to GAD65 puncta was determined in percentage. The average number of 15 images, per condition, was calculated for 3 independent experiments. Likewise, the number of GAD65 puncta per unit length of axon was determined by selecting the option Trace Region on MetaMorph to delineate segments of axons with at least 100 µm, and measuring the number of GAD65 puncta on the image previously submitted to a threshold, using Integrated Morphometric Analysis. The length of each axonal segment was determined selecting the Multi-line tool and uniting consecutive puncta along the delineated neurite. This procedure was repeated in each field of view, for 10–15 images per condition, in each of the 3 independent neuronal preparations.

### Determination of the viability of GABAergic neurons with Hoescht 33342

Determination of cell viability was performed by fluorescence microscopy, using the indicator Hoechst 33342 as previously described [40]. The cells were stimulated with 125 µM glutamate for 20 min, in Neurobasal medium supplemented with the GABA transporter inhibitor SKF89976 (10 µM). After the excitotoxic insult hippocampal neurons were further incubated in culture conditioned medium supplemented with 10 µM SKF89976 for 12 h. Incubation of the cells with SKF89976 during stimulation with glutamate and after the excitotoxic insult prevents the depletion of GABA through reversal of the plasma membrane transporter [41]. GABAergic neurons were stained using an anti-GABA polyclonal antibody (see above), and the nuclear morphology was assessed through staining with Hoechst 33342. Analysis of the nuclear morphology was limited to GABAergic neurons, stained with the anti-GABA antibody.

### Measurement of metabolic activity with CellTiter-Glow

Rat hippocampal neurons cultured in 384 micro-titter plates, coated with poly-D-lysine, at a density of 91.6×10^3^ cells/cm^2^ were incubated with 125 ìM glutamate in supplemented Neurobasal medium, for 20 min at 37°C. After stimulation with glutamate, the cells were further incubated with the original culture medium, for 14 h at 37°C. The viability of the cells was then measured by analysing the levels of ATP as an indicator of cellular metabolic activity, using CellTitter-Glo (Promega) according to manufacturer's instructions. Briefly, in each well, 50 ìl of PBS/CellTitter-Glo (1∶1) were dispensed with a Multidrop 384 stacker (Titertek), after removal of the growth media, at room temperature. The plate was then placed on an orbital shaker for 2 minutes, at maximum speed and further incubated for 10 minutes at room temperature, without shaking. Luciferase luminescence was measured immediately afterwards, using an Acquest plate reader (Molecular Devices).

### Statistical Analysis

Statistical analysis was performed using one-way ANOVA analysis of variance followed by the Bonferroni test, or using the Student's *t* test, as indicated in the figure captions.

## Results

### Excitotoxic damage of cultured GABAergic hippocampal neurons

Dissociated cultures of hippocampal neurons contain glutamatergic and GABAergic neurons, expressing the vesicular glutamate transporter 1 (VGLUT1) and glutamic acid decarboxylase, respectively [42]. Immunocytochemistry experiments using antibodies against VGLUT1+VGLUT2 (Fig. 1D) and against GABA (Fig. 1E) showed that 65% of the cells present in hippocampal cultures are glutamatergic and 5% are GABAergic, respectively (Fig. 1A).

Excitotoxic stimulation of cultured hippocampal neurons with 125 µM glutamate for 20 min reduced cell viability as determined using the CellTiter-Glo Luciferase chemiluminescence assay, a method based on the quantification of the ATP present in the cells. Luciferase activity was reduced to 48% of the control 14 h after the toxic insult (Fig. 1B), in agreement with the results obtained in experiments where cell survival was determined using fluorescence microscopy with the indicator Hoechst 33342 [40]. Under these conditions damaged hippocampal neurons display an apoptotic-like morphology. Since GABAergic neurons represent a minor fraction of the cells present in the cultures, we have specifically assessed the effects of excitotoxic stimulation with glutamate on the neuronal population displaying GABA immunoreactivity. In these experiments the cells were stimulated with glutamate in the presence of the GABA transporter inhibitor SKF89976 in order to prevent the release of the neurotransmitter through reversal of the plasma membrane transporter [41]. The number of GABAergic cells displaying apoptotic-like morphology 12 h after glutamate stimulation was about 54% (Fig. 1C).

### Excitotoxicity-induced cleavage of GAD and down-regulation of gene expression

**Figure 2:**
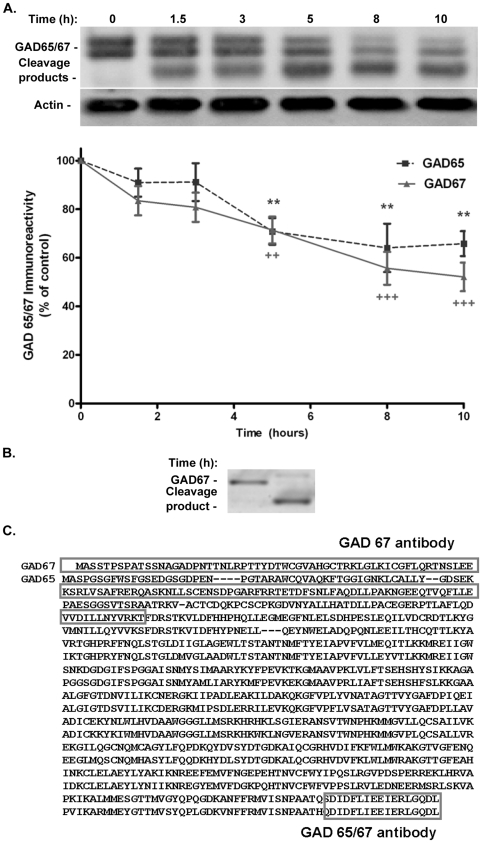
Glutamate excitotoxicity induces a time-dependent decrease in GAD65 and GAD67 protein levels in cultured hippocampal neurons. Neurons were stimulated with 125 µM glutamate, for 20 min, and further incubated in culture conditioned medium for the indicated period of time. Full length GAD 65/67 protein levels were determined by Western Blot with an antibody that recognizes both isoforms. Control protein levels of GAD65/67 were set to 100%. Actin was used as loading control (A). Panel A shows a representative experiment and mean±SEM of 9 independent experiments. The cleavage of GAD67 was also analysed with an antibody directed against amino acids 70–130 of this isoform (B). In this case the results obtained under control conditions were compared with the immunoreactivity in extracts prepared 14 h after the toxic insult. The amino acid sequence of GAD65 (lower sequence) and 67 (top sequence) are aligned in panel C, which also show the binding sites for the antibodies used in this study. Statistical analysis was performed using one-way ANOVA, followed by Bonferroni's multiple comparison test. **p<0.01; ***p<0.001.

To test the effect of glutamate stimulation on glutamic acid decarboxylase, a marker of GABAergic neurons, GAD protein levels were evaluated after the excitotoxic insult, using an antibody that recognizes both forms of the enzyme in a common C-terminal region (Fig. 2C). Under control conditions the antibody allows identifying the two GAD isoforms, with 67 kDa and 65 kDa. Glutamate stimulation induced a time-dependent decrease in the abundance of both isoforms, and this effect was correlated with the upregulation of a truncated form with an apparent molecular mass of 55–58 kDa (Fig. 2A). The truncated form still bound the C-terminus directed antibody, but no smaller immunoreactive forms of GAD were detected in the blots (not shown). These results indicate that glutamate-induced cleavage of the two GAD isoforms occurs at the N-terminal region and gives rise to truncated forms with similar apparent molecular weights.

In order to further characterize the cleavage of GAD under excitotoxic conditions, we tested a GAD67 specific antibody that binds its N-terminus (amino acids 17–130). The immunoreactivity pattern in extracts prepared from cells incubated for 14 h after the toxic insult with glutamate was similar to that obtained using the antibody directed against the C-terminal region of GAD (Fig. 2B). This indicates that GAD67 is cleaved before amino acid 130.

**Figure 3:**
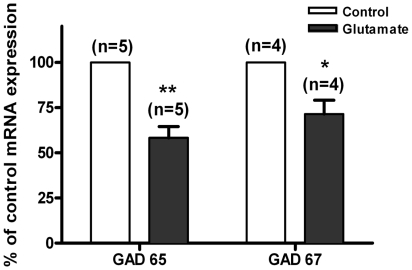
Glutamate excitotoxicity decreases GAD65/67 mRNA. Gene expression was analysed in cultured hippocampal neurons (7 DIV) exposed or not to 125 µM Glutamate, for 20 min, and then returned to the original culture medium for 4 h. For the reverse transcription reaction 1 µg of total RNA was used. The results were normalized with two internal control genes, GAPDH and Tubulin. Data are presented as mean±SEM of four to five independent transcription reactions, performed in independent preparations. Statistical analysis was performed using Student's *t*-test. *p<0.05; **p<0.01.

Besides its effect in inducing the cleavage of GAD, excitotoxic stimulation with glutamate may also have delayed effects on GAD by acting at the transcription level. This was tested by Real-Time PCR, in cells subjected to excitotoxic stimulation with glutamate for 20 min and further incubated in culture medium for 4 h. Under these conditions there was a 58% and 71% downregulation of GAD65 and GAD67 mRNA, respectively, relative to unstimulated cells (Fig. 3).

### Proteasome inhibitors protect GAD65/67 from cleavage under glutamate-induced excitotoxicity in hippocampal neurons

**Figure 4:**
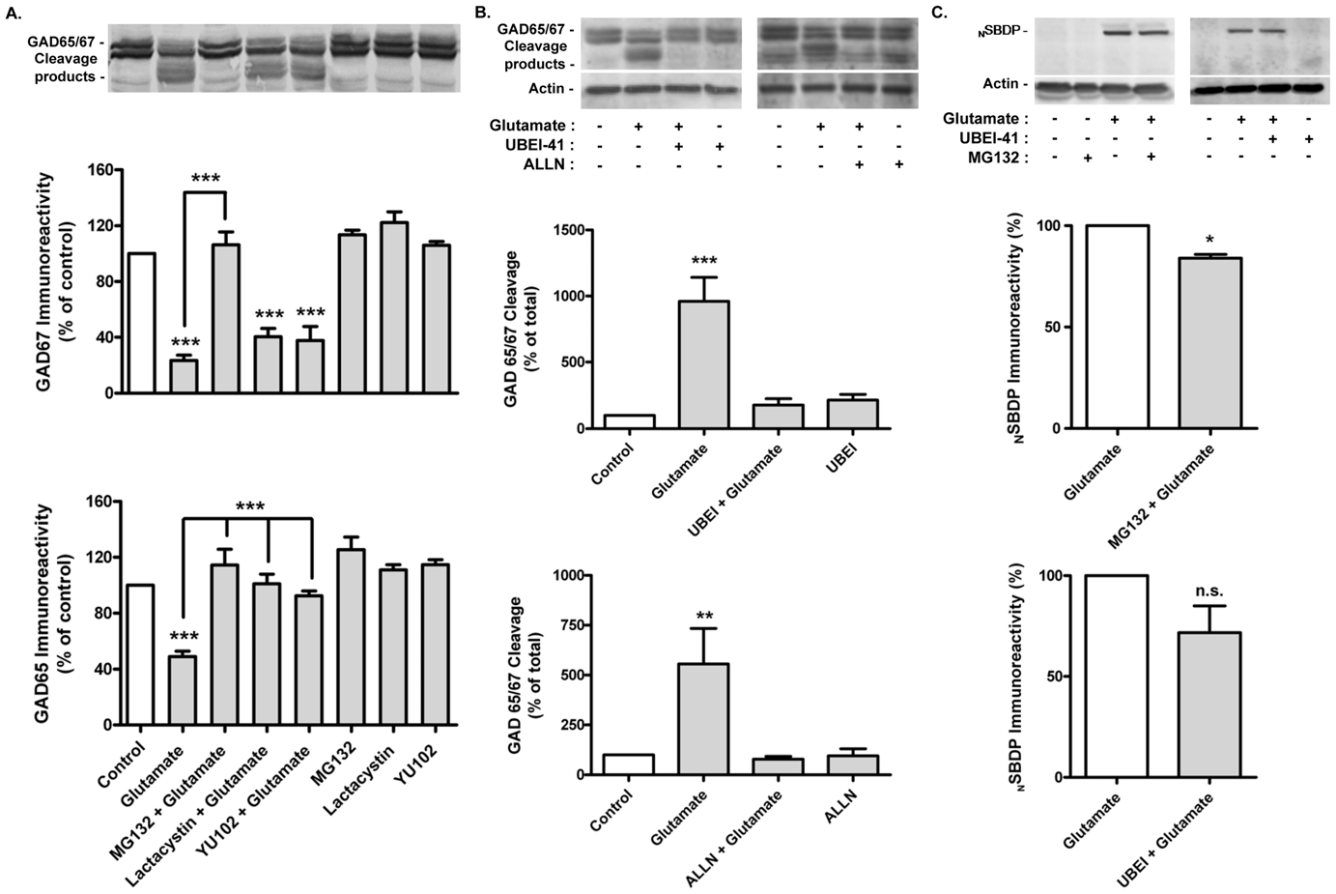
Proteasome and ubiquitin-activating enzyme (E1) inhibitors prevent glutamate–induced GAD65/67 cleavage. Cultured hippocampal neurons were pre-incubated or not with 50 µM of UBEI-41 (E1 inhibitor), for 1h, or with the proteasome inhibitors MG132 (1 µM), lactacystin (10 µM) or YU102 (10 µM), for 30 min, before excitotoxic stimulation with glutamate (125 µM), for 20 min. The cells were further incubated in culture conditioned medium (with or without chemical inhibitors) for 5h, and the GAD65/67 immunoreactivity was assessed by western blot (A and B). Incubation with the calpain inhibitor ALLN (10 µM) was performed under the same conditions. The average results in (A) represent the changes in GAD65 or GAD67 immunoreactivity. In panel (B) GAD cleavage was calculated as a percentage of the total enzyme content (GAD65/67). When calpain activity was evaluated through formation of N-terminal spectrin breakdown products (_N_SBDPs) the cells were incubated for 30 min after the toxic insult (C). The effect of MG132 and UBEI-41 on calpain activation is expressed as a percentage of the activity measured in the absence of the protease inhibitors. Results are means±SEM of 3–4 different experiments, performed in independent preparations. Statistical analysis was performed using one-way ANOVA, followed by Bonferroni's Multiple Comparison Test (A and B) or the Student's *t* test (C). ***p<0.001.

Multiple proteolytic systems have been shown to participate in the cleavage of GAD under excitotoxic conditions, including calpains and cathepsins [22]–[25]. Despite the key role of the ubiquitin-proteasome system (UPS) in protein degradation in the CNS, no studies have addressed its role in the down-regulation of full-length GAD isoforms under excitotoxic conditions. To test for the effect of inhibiting different proteolytic activities of the proteasome, we used the chymotrypsin-like activity directed inhibitor MG132 and the post-glutamyl peptide hydrolyzing-activity (PGPH) directed inhibitor YU102. We also tested the effect of lactacystin which shows a slight preference for the trypsin-like and caspase-like activities [43]. MG132 is a synthetic peptide aldehyde that binds reversibly to the 20S proteasome active site forming a covalent hemiacetal adduct [44], [45]. The effect of proteasome inhibitors was tested 5 h after the toxic insult with glutamate since long incubation periods with these compounds causes neuronal cell death [46], [47]. MG132 abrogated glutamate-induced cleavage on both isoforms of GAD, as determined 5 h after the toxic insult (Fig. 4A). Lactacystin is a *Streptomyces lactacystinaeus* metabolite that targets the 20S proteasome by an irreversible modification of the amino terminal threonine of β-subunits, while YU102 is a α′, β′-epoxyketine, the only peptidyl-glutamylpeptidehydrolyzing (PGPH)-specific peptide used in this study [45], [48]. Both YU102 and lactacystin inhibited glutamate-evoked GAD65 cleavage, but were without effect on GAD67 (Fig. 4A).

To further characterize the role of the UPS in the glutamate-evoked cleavage of GAD we tested the effect of the ubiquitin-activating enzyme (E1) inhibitor, UBEI-41 [49]. It has been assumed that only a single activating enzyme for ubiquitin exists, which operates at the initial step of the ubiquitin-proteasome pathway. Therefore, if the UPS plays a role in the cleavage of GAD, inhibitors of E1 should abrogate the excitoxicity-induced cleavage of the GAD. Accordingly, inhibition of the ubiquitin-activating (E1) enzyme prevented the effect of excitotoxic stimulation on the cleavage of GAD67 and GAD65 (Fig. 4B).

In previous studies the cleavage of GAD under excitotoxic conditions was found to be abrogated by calpain inhibitors [22]–[25]. Furthermore, recombinant GAD65 and 67 are cleaved in vitro by calpain [22], [23]. Accordingly, incubation of hippocampal neurons with ALLN, a chemical inhibitor that targets preferentially calpains when used at lower concentrations, prevented the glutamate-induced cleavage of GAD65/67 (Fig. 4B). Therefore, we determined whether inhibition of calpains could account for the effect of the UPS inhibitors on glutamate-induced cleavage of GADs. Activation of calpain was measured by western blot, using an antibody that binds specifically to the product resulting from the cleavage of spectrin by calpains (SBDPs) [37], [38], [50]. Glutamate stimulation increased the formation of N-terminal SBDPs (_N_SBDPs), and this effect was only slightly inhibited by MG132 (16.6%; p<0.05) and by UBEI-41 (28.3%; p>0.05) (Fig. 4C). These results indicate that calpain inhibition is not likely to account for the effects of MG132 and UBEI-41 on glutamate-evoked cleavage of GAD.

### GAD65/67 interact with ubiquitinated proteins in primary hippocampal cultures

**Figure 5:**
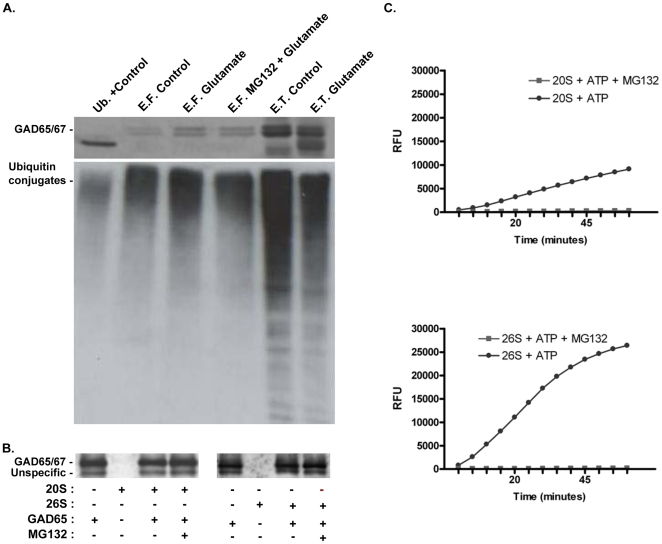
GAD 65/67 are captured with an anti-ubiquitin antibody in hippocampal cultures. (A) Cultured hippocampal neurons were stimulated or not with 125 µM glutamate for 20min, in the presence or in the absence of 10 µM MG132, and the cells were further incubated with culture conditioned medium for 4h before preparation of the extracts. In the top panel, mono- and poly-ubiquitinylated proteins were isolated using the UbiQapture™-Q Kit, and the eluted fraction [46] was subjected to western blot, using an antibody against GAD65/67. GAD65/67 total immunoreactivity in the extracts prepared from control cells and from hippocampal neurons stimulated with glutamate is shown on the right (E.T.). The left lane was loaded with a control provided in the kit, consisting in ubiquitinated protein lysate. The same membranes were probed for mono- and poly-ubiquitin using an antibody included in the kit (A, middle). (B) Human recombinant GAD65 (0.75 µg) were incubated with 20/26S proteasomes (1.5 µg) for 2h at 37°C with or without MG132. The extracts were then probed with GAD65/67 antibody. The activity of the 20S/26S proteasomes used in the experiments was confirmed using the fluorogenic substrate suc-LLVY-MCA. The increase in fluorescence resulting from the cleavage of the substrate was measured in relative fluorescence units (RFU) (C). The results of the capture of ubiquitinated proteins and the assay of the recombinant GAD65 cleavage are representative of two and three independent experiments, respectively.

The results shown above suggest that the proteasome plays a direct role in GAD67 cleavage under excitotoxic conditions. In particular, the inhibition of GAD67 cleavage by the E1 inhibitor (Fig. 4C) suggests that the enzyme is ubiquitinated before cleavage by the proteasome. To test for the possible ubiquitination of GADs we used the UbiQapture™-Q Kit which allows isolating both mono- and poly-ubiquitinylated proteins (and their binding partners), independent of lysine residue chain linkage. Ubiquitinated proteins (and their binding partners) were isolated from extracts of hippocampal neurons stimulated or not with glutamate (with or without MG132), and the results were analysed by Western Blot with an anti-GAD65/67 antibody. GAD65/67 was immunoprecipated in similar amounts in all experimental conditions tested, but stimulation with glutamate in the presence or in the absence of MG132 increased the capture of GAD (Fig. 5A, top panel). However, in all experimental conditions the mobility of the immunoprecipitated GAD65/67 was the same as the mobility of the protein present in extracts directly loaded on the gel, suggesting that there is no change in GAD ubiquitination following glutamate stimulation. Taken together these results suggest that GAD67 interacts with another protein(s) that is ubiquitinated, and capture of this protein by an anti-ubiquitin antibody allows co-purification of the enzyme. The increased co-immunopurification of GAD67 in extracts from cells stimulated with glutamate may suggest that the excitotoxic insult increases the ubiquitination of the GAD67 interacting protein(s).

Since the 20S proteasome is able to cleave substrates without ubiquitination [51], [52], an in vitro system was used to determine whether this could account for the observed inhibitory effect of MG132 on the excitotoxicity-induced cleavage of GAD65. Recombinant GAD65 was incubated with 20S and 26S proteasomes using the protocol previously described [51], which allowed characterizing the ubiquitin- and ATP-independent cleavage of YB-1 (a DNA/RNA-binding nucleocytoplasmic shuttling protein) by the 20S proteasome in vitro. No cleavage of recombinant GAD65 was observed following incubation with the 20S or 26S proteasome (Fig. 5B), suggesting that this GAD isoform does not undergo a ubiquitin-independent proteasomal cleavage, as described for YB-1. Control experiments using fluorogenic substrates showed that the 20S and 26S proteasome preparations were active (Fig. 5C), further suggesting that the proteasome does not act directly on GAD65.

### GADs cleavage is correlated with decreased enzyme activity and changes the subcellular distribution

**Figure 6:**
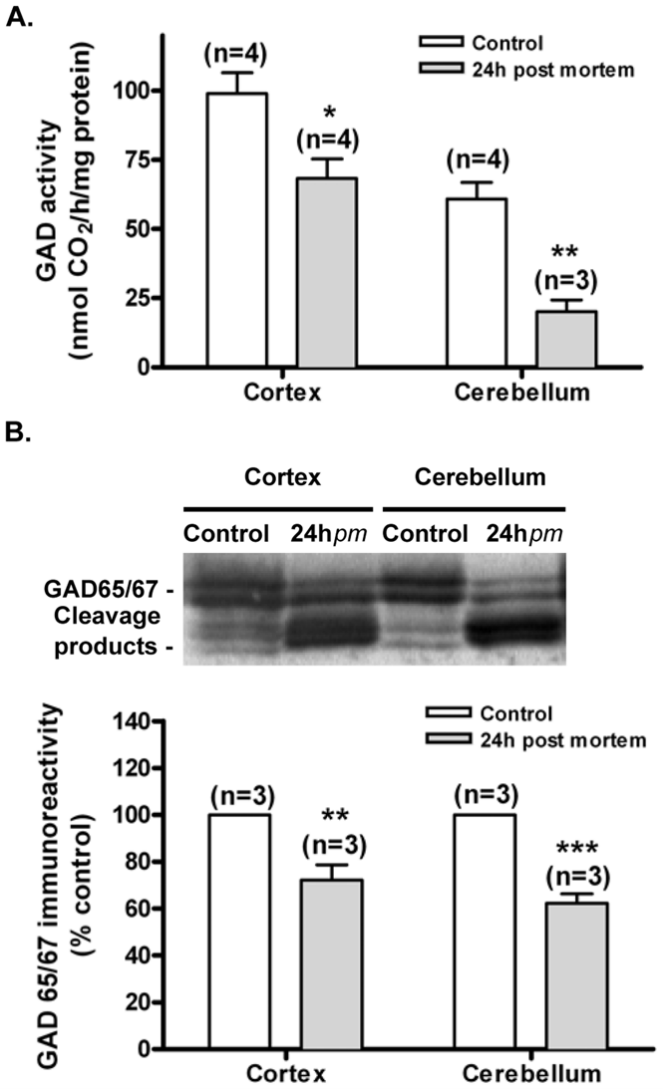
Excitotoxicity-induced decrease in GAD65/67 activity. Heads of adult Wistar rats were decapitated and processed immediately or kept for 24 h at room temperature. The extracts were used for both GAD activity measurements and Western Blot analysis. GAD activity was determined using a trapping technique for radiolabelled [^14^C]CO_2_ brought by GAD65/67 activity, and was expressed as nmol CO_2_/hr/mg of protein (A). Full-length GAD65/67 protein levels from the same extracts were determined by Western Blot using an anti-GAD65/67 antibody, and control protein levels of GAD65/67 were set to 100% (B). Data are presented as mean±SEM of 3 to 4 independent experiments. Statistical analysis was performed using Student's *t*-test. *p<0.05; **p<0.01: ***p<0.001.

Since GADs play a key role in the synthesis of GABA from glutamate, we investigated how the cleavage of the enzyme affects its activity. The assay of GAD activity using the [^14^C]CO_2_ trapping method requires the use significant amounts of protein that cannot be obtained using hippocampal cultures. Therefore, the effect of GAD cleavage on the activity of the enzyme was investigated using brain tissue from decapitated rats. Previous studies have shown that under these conditions GAD is cleaved with a pattern similar to that observed under excitotoxic conditions, particularly in the cerebellum and in the cerebral cortex [53]. The post-mortem cleavage of GAD65 and GAD67 in these brain regions was confirmed in the present study (Fig. 6B), and 24 h after death there was a decrease in the total full length GAD protein levels both in the cerebellum and in the cerebral cortex (Fig. 6A). At this time point the activity of GAD was decreased to 68.8% in the cerebral cortex and to 33.1% in the cerebellum, while the total amount of full-length protein was reduced to 73% and 58%, respectively. The total GAD protein levels (full-length + cleaved protein) at 24 h post-mortem was not significantly different from the amount of protein detected under control conditions (see representative western blot in the top panel of Fig. 6B).

**Figure 7:**
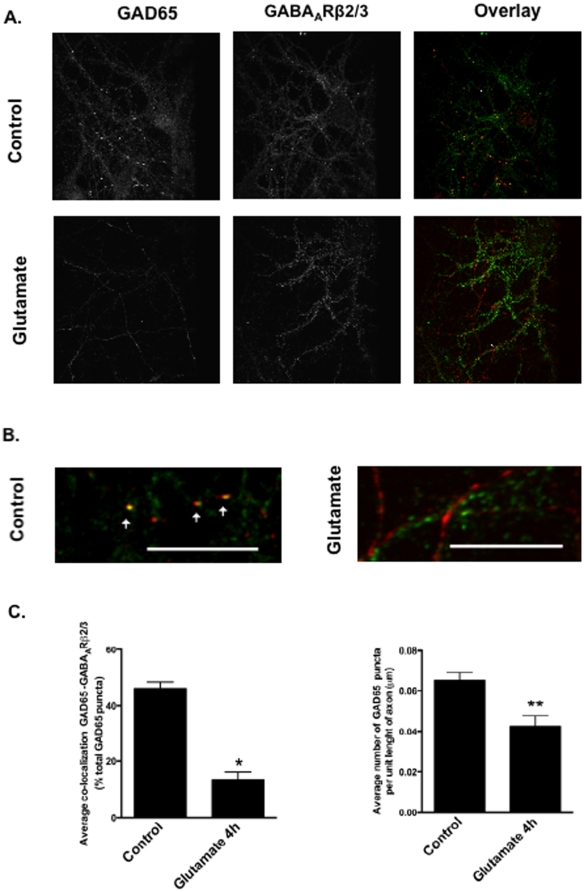
Glutamate changes the subcellular distribution of GAD65 along neurites. 10 DIV hippocampal neurons were incubated with or without glutamate (125 µM) for 20 min, and then returned to the original culture medium for 4 h.(A) Cells were fixed, permeabilized and probed with specific antibodies to GAD65 and GABA_A_ receptor subunits β2/3. (B) Arrows indicate GAD65 clustering (red), a pattern that is changed in glutamate treated cells. Images are representative of three different experiments performed in independent preparations. Images in (B) show colocalization of the immunoreactivity for GAD65 (red) and GABA_A_ receptor subunits β2/3 (green) under control conditions, and the redistribution of GAD65 in the axons of hippocampal neurons subjected to excitotoxic conditions. The scale bar corresponds to 10 µm. (C) Quantification of the images for the colocalization of GAD65 and GABA_A_ subunits β2/3, expressed as percentage of total GAD65 puncta (left), and the average number of GAD65 puncta per axon unit length (right). Data are presented as mean±SEM of 3 independent experiments performed in different preparations. Statistical analysis was performed using Student's *t*-test. *p<0.05; **p<0.01.

GAD65 is anchored to synaptic vesicles through its N-terminus [54], [55]. Since glutamate stimulation cleaves GAD near the N-terminal region, we hypothesized that the cleavage of the enzyme could affect its sub-cellular localization. Under control conditions GAD65 displays a partially punctate distribution along neurites (Fig.7A, arrowheads), but this pattern is altered 4 h after excitotoxic stimulation with glutamate. Under the latter conditions some neurites show a more homogeneous distribution of GAD65, diffuse along the neuronal processes (Fig. 7A, B), and the number of GAD65 puncta is significantly reduced in comparison to the control conditions (Fig. 7C). Colocalization of GAD65 with the β2/3 GABA_A_ receptor subunits was also significantly decreased (Fig. 7C), showing a loss of synaptic distribution of GAD65 under excitotoxic conditions.

## Discussion

Previous studies have shown the cleavage of the glutamic acid decarboxylase isoforms GAD65 and GAD67 under excitotoxic conditions [23], [25], and pointed out a key role for calpains in this process [22]–[24]. In this work we show that the activity of E1 ubiquitin ligase and the proteasome are required for glutamate-evoked cleavage of GAD67, although no clear evidences were obtained showing a direct ubiquitination of the enzyme. Furthermore, cleavage of GAD65/67 was found to decrease enzyme activity and changed the characteristic punctate distribution of GAD65 along neurites. Both effects are likely to downregulate the activity of GABA as a neurotransmitter under excitotoxic conditions.

### Glutamate-induced cleavage of GAD protein levels and downregulation of mRNA

Excitotoxic stimulation of cultured hippocampal neurons induced the cleavage of GAD65 and 67 by a mechanism sensitive to the calpain inhibitor ALLN, similarly to what was observed in neuronal cultures prepared from the whole brain or from the cerebral cortex [23], [24]. The full-length proteins were cleaved into a truncated form with approximately 55–58 kDa, which was detected by an antibody directed against the N-terminal of GAD65 and GAD67. Since no immunoreactive bands with low apparent molecular weight were identified, the results indicate that both GAD isoforms are cleaved in a sequence close to the N-terminal region of the proteins. Accordingly, an antibody directed against amino acids 17–130 of GAD67 also detected the cleavage product of the enzyme, showing that the cleavage site is located before amino acid 130. The sequence after amino acid 100 in GAD67 shows high homology with GAD65 (Fig. 2C), and this explains the similarity in the apparent molecular weight of the cleavage products of GAD65 and GAD67. Much of the available evidences suggest that the N-terminal segment of GAD is exposed and flexible [56], and this may make this region available for cleavage by proteases. In vitro studies showed that recombinant human GAD67 lacking the first 70 or the first 90 amino acids is not cleaved by calpain, in contrast with the full length protein [22], suggesting that under excitotoxic conditions GAD67 may be cleaved between amino acids 90 and 130. If this is the case, the dimerization of GADs required for their activity is likely not affected by enzyme cleavage since dimer formation occurs through interaction of C-terminal portions of GAD molecules [56]. GAD is a pyridoxal 5′-phosphate (PLP)-dependent enzyme, but the co-enzyme binding site is not contained within the N-terminal regions [57]. Therefore, changes in PLP binding are not likely to account for the changes in GAD activity following enzyme cleavage.

In addition to the cleavage of GAD we also observed a decrease in the mRNA levels for both isoforms of the enzyme in hippocampal neurons subjected to an excitotoxic insult with glutamate (Fig. 3). This is likely to limit the de novo synthesis of GAD, which could otherwise compensate for the observed dowregulation of the full-length protein. It remains to be determined whether the observed decrease in the mRNA for GAD is due to a reduction in transcription activity and/or to an active degradation of the existing transcripts. The rapid down-regulation of GAD mRNA following excitotoxic stimulation of cultured hippocampal neurons contrasts with the delayed effects of ischemic injury on GAD67 mRNA [58]; unilateral ischemic lesions of the frontoparietal cortex in adult rats increased GAD67 mRNA levels in the striatum, lasting up to 3 months after surgery. Inhibition of NMDA receptors also downregulated GAD mRNA in various brain regions, starting at day 2 after treatment [59], suggesting that glutamate receptors are directly coupled to the activation of GAD67 expression.

### UPS system activity is essential for excitotoxicity-induced GAD cleavage

Previous studies have shown that calpain inhibitors fully block [23] [or inhibit to a great extent [24]] the glutamate evoked cleavage of GAD65/67 (see also Figure 4B), and in vitro experiments showed that calpains cleave recombinant GAD67 [22]. Taken together these evidences strongly suggest that calpains play a key role (if not exclusive) in GAD cleavage under excitotoxic conditions. Surprisingly, we observed that inhibition of the proteasome with MG132, lactacystin or YU102 fully abrogated the cleavage of GAD67 in hippocampal neurons subjected to excitotoxic conditions, and MG132 had the same effect on the cleavage of GAD65. Furthermore, inhibition of the ubiquitin-activating enzyme (E1) with UBEI-41 also prevented the cleavage of GAD67 and GAD65. The effects of MG132 and UBEI-41 cannot be attributed to inhibition of calpains since the inhibitors had a small (MG132) or no effect (UBEI-41) on the glutamate-evoked calpain activation, as determined by measuring the formation of spectrin breakdown products (Fig. 4C). Since the molecular weight of the GAD truncated forms observed in the present studies is similar to that observed in previous studies where calpains were shown to participate in the cleavage of the enzyme, it is likely that the UPS and calpains act in a co-ordinated manner to cleave GADs. If this is the case, the UPS is likely to act upstream of calpains since no evidences were found for a direct effect of proteasome in GAD cleavage.

Interaction between calpains and the UPS is also physiologically relevant in other scenarios. Sequential activity of calpains and the UPS has been proposed to contribute to the degradation of myofibrils; calpains release myofibrils form the contractile apparatus, which allows initiating ubiquitination and degradation by the proteasome [60], [61]. Also, degradation of IκB through phosphorylation-dependent ubiquitination or following cleavage by calpains is thought to release NFκB, and the transcription factor migrates to the nucleus where it binds DNA [62]–[64]. There are reports suggesting that proteasome inhibition could be neuroprotective after stroke [31], [65], namely through stabilization of IκB and thereby preventing NF-κB activation. Inhibition of calpains can also provide functional neuroprotection in various animal models of cerebral ischemia [66].

### The role of ubiquitination in GADs cleavage

The glutamate-evoked cleavage of GAD65/67 was sensitive to proteasome inhibition, but incubation of GAD65 with the 20S proteasome did not give rise to the cleavage product of the enzyme. Although some proteins are cleaved by the 20S proteasome without ubiquitination [51], [52], this is not the case of GAD65.

Inhibition of the E1 ubiquitinating enzyme also abrogated the cleavage of GAD65/67 under excitotoxic conditions, indicating that protein ubiquitination plays a key role in the process. Separation of mono and poly-ubiquitinated proteins with the UbiQapture™-Q Kit allowed recovering GAD65 and GAD67, but although there was an increase in the amount of GAD isolated with the kit under excitotoxic conditions the apparent molecular weight of the proteins isolated was similar to that observed in whole cell extracts. This strongly suggests that GAD is not ubiquitinated, but instead interacts with a protein which state of ubiquitination is increased following excitotoxic stimulation with glutamate. This may be related with an increase in ubiquitin mRNA transcripts, as observed following ischemia [67], [68], with an impairment of the proteasome activity [29], [30], [69], and/or to a signalling cascade induced by excitotoxicity that may ultimately leads to the ubiquitination of the GAD binding partner. Excitotoxicity also regulates the NF-κB transcription factor after cerebral ischemia through ubiquitination and degradation of its binding partner IκB [70]. Similarly, the cleavage of GAD may follow the increase in ubiquitination and degradation of a binding partner, which may allow cleavage of the enzyme by calpains. This hypothesis explains the effect of both E1 inhibition and proteasome inhibition on GAD cleavage, and the results showing no apparent ubiquitination of the enzyme (present work), and the role of calpains [24]. The GAD binding partner that may be involved in the regulation of the protein under excitotoxic conditions remains to be identified.

Interestingly, the proteasome inhibitors showed differential effects on GAD65 and GAD67 cleavage induced by excitotoxic stimulation with glutamate. MG132 abrogated the cleavage of both GAD isoforms, in contrast with YU102 and lactacystin which were only effective against the cleavage of GAD67. The difference in the effects of the inhibitors tested may be due to their specificities: MG132 and YU102 act preferentially on the cheymotrypsin-like and caspase-like activities of the proteasome, respectively, whereas lactacystin targets preferentially the trypsin-like and caspase-like activities [43], [48]. Assuming that under excitotoxic conditions the proteasome targets a GAD binding partner before cleavage of the enzymes by calpains, these results suggest that the proteasome substrates bound to each of the GAD isoforms are distinct.

### Alterations of GADs activity and localization under excitotoxic conditions

The decrease in GADs activity observed in *post mortem* cerebral cortices to 74% was correlated with a decrease to 73% of the full-length protein found in the extracts analysed by Western Blot; in the cerebellum the GAD activity decrease to 48% in *post mortem* extracts relative to control, and a down-regulation of the full-length protein to 58% was observed. This decrease in enzyme activity following N-terminal cleavage is in agreement with previous results obtained using a similar experimental paradigm [53] and with the effect of calpain cleavage at the N-terminal region on the activity of recombinant GAD67 [22]. In contrast, truncation of the N-terminal region of recombinant GAD65 increased enzyme activity [71] and trypsin cleavage of recombinant GAD65 and GAD67 at their N-terminal region was shown to increase enzyme activity [56]. The difference between the effects observed in brain extracts and in *in vitro* experiments may be due to interaction of GAD with regulatory proteins (see below), which are absent when recombinant proteins are used. Post-translational modifications of GAD, such as phosphorylation, may also contribute to the differences in the effect of N-terminal cleavage on the activity of the enzyme measured in brain extracts or using recombinant protein [72]. A decrease in enzyme activity in neurons subjected to excitotoxic conditions may activate compensatory mechanisms in surviving neurons since the expression of GAD is regulated by the abundance of GABA by a mechanism independent of the activation of GABA receptors [73], [74]. However, within the time frame analysed after the excitotoxic insult we found no evidences for an upregulation of GAD protein levels from de novo protein synthesis (Fig. 2).

In this work we also found that excitotoxic stimulation of cultured hippocampal neurons changes the subcellular distribution of GAD65, with a loss of protein clustering along neurites. This is in agreement with the results showing a role for palmitoylation of Cys30 and Cys45 in GAD65 in the post-Golgi trafficking of the protein to presynaptic clusters [75], [76]. The N-terminal truncation under excitotoxic conditions is likely to separate this targeting sequence from the catalytic domain of GAD65, dissociating the enzyme from synaptic vesicles as suggested in the results of the immunocytochemistry experiments shown in Fig. 7. Furthermore, the decrease in colocalization of GAD65 and the β2/3 GABA_A_ receptor subunits suggests that the cleaved protein becomes more diffuse, moving away from the synapse.

It was proposed that association of GAD with membranes and the anchoring of the enzyme to synaptic vesicles occur first through formation of a complex with the heat shock protein 70 family member HSC70 (heat shock cognate 70), followed by interaction with cysteine string protein (CSP), an integral protein of the synaptic vesicle [54]. Cleavage or degradation of the GAD anchoring proteins may release the enzymes anchored to synaptic vesicles and may contribute to change the subcellular distribution of the enzyme under excytotoxic conditions. If the N-terminal region of GAD65 plays a role in the interaction with the anchoring proteins, the cleavage of the enzyme under excitotoxic conditions may also explain the observed changes in immunoreactivity after the toxic insult with glutamate. The interaction of GAD with HSC70 and synaptic vesicles also promotes the activity of the enzyme [54]. The release of GAD65 from synaptic vesicles that may occur under excitotoxic conditions would explain, at least in part, the decrease in enzyme activity observed in cerebellar and cerebrocortical extracts containing cleaved GAD.

The anchoring of GAD65 to synaptic vesicles through interaction with the vesicular GABA transporter may allow coupling the synthesis of GABA to the packaging of the neurotransmitter into the vesicles [17]. The cleavage of the N-terminal region of GAD65 and the consequent dissociation of the enzyme from synaptic vesicles and from the synapse may decrease the accumulation of GABA in the vesicles and, therefore, may deregulate GABAergic synapses. This is particularly relevant considering that GAD65 is the isoform responsible for the synaptically released GABA [77].

In conclusion, we showed that excitotoxic conditions lead to the cleavage of GAD65/67 in cultured hippocampal neurons in a UPS-dependent manner. GAD cleavage decreased enzyme activity and changed the subcellular distribution of the 65KDa isoform, which should decrease GABA production and may affect the accumulation of the neurotransmitter in synaptic vesicles.

## References

[1] Lipton SA, Rosenberg PA (1994). Excitatory amino acids as a final common pathway for neurologic disorders.. N Engl J Med.

[2] Choi DW (1988). Glutamate neurotoxicity and diseases of the nervous system, Neuron.

[3] Gardoni F, Di Luca M (2006). New targets for pharmacological intervention in the glutamatergic synapse.. Eur J Pharmacol.

[4] Lewen A, Li GL, Olsson Y, Hillered L (1996). Changes in microtubule-associated protein 2 and amyloid precursor protein immunoreactivity following traumatic brain injury in rat: influence of MK-801 treatment.. Brain Res.

[5] Carvalho AP, Ferreira IL, Carvalho AL, Duarte CB (1995). Glutamate receptor modulation of [^3^H]GABA release and intracellular calcium in chick retina cells.. Ann N Y Acad Sci.

[6] Saransaari P, Oja SS (2008). GABA release under normal and ischemic conditions.. Neurochem Res.

[7] Schwartz-Bloom RD, Sah R (2001). gamma-Aminobutyric acid_A_ neurotransmission and cerebral ischemia.. J Neurochem.

[8] Vemuganti R (2005). Decreased expression of vesicular GABA transporter, but not vesicular glutamate, acetylcholine and monoamine transporters in rat brain following focal ischemia.. Neurochem Int.

[9] Baliova M, Knab A, Franekova V, Jursky F (2009). Modification of the cytosolic regions of GABA transporter GAT1 by calpain.. Neurochem Int.

[10] Bevers MB, Neumar RW (2008). Mechanistic role of calpains in postischemic neurodegeneration.. J Cereb Blood Flow Metab.

[11] Martin DL, Rimvall K (1993). Regulation of gamma-aminobutyric acid synthesis in the brain, J Neurochem.

[12] Erlander MG, Tillakaratne NJ, Feldblum S, Patel N, Tobin AJ (1991). Two genes encode distinct glutamate decarboxylases.. Neuron.

[13] Sheikh SN, Martin SB, Martin DL (1999). Regional distribution and relative amounts of glutamate decarboxylase isoforms in rat and mouse brain.. Neurochem Int.

[14] Hsu CC, Thomas C, Chen W, Davis KM, Foos T (1999). Role of synaptic vesicle proton gradient and protein phosphorylation on ATP-mediated activation of membrane-associated brain glutamate decarboxylase.. J Biol Chem.

[15] Nathan B, Floor E, Kuo CY, Wu JY (1995). Synaptic vesicle-associated glutamate decarboxylase: identification and relationship to insulin-dependent diabetes mellitus.. J Neurosci Res.

[16] Reetz A, Solimena M, Matteoli M, Folli F, Takei K (1991). GABA and pancreatic beta-cells: colocalization of glutamic acid decarboxylase (GAD) and GABA with synaptic-like microvesicles suggests their role in GABA storage and secretion.. EMBO J.

[17] Jin H, Wu H, Osterhaus G, Wei J, Davis K (2003). Demonstration of functional coupling between gamma -aminobutyric acid (GABA) synthesis and vesicular GABA transport into synaptic vesicles, Proc Natl Acad Sci U S A.

[18] Wu H, Jin Y, Buddhala C, Osterhaus G, Cohen E (2007). Role of glutamate decarboxylase (GAD) isoform, GAD65, in GABA synthesis and transport into synaptic vesicles-Evidence from GAD65-knockout mice studies.. Brain Res.

[19] Asada H, Kawamura Y, Maruyama K, Kume H, Ding R (1996). Mice lacking the 65 kDa isoform of glutamic acid decarboxylase (GAD65) maintain normal levels of GAD67 and GABA in their brains but are susceptible to seizures.. Biochem Biophys Res Commun.

[20] Kaufman DL, Houser CR, Tobin AJ (1991). Two forms of the gamma-aminobutyric acid synthetic enzyme glutamate decarboxylase have distinct intraneuronal distributions and cofactor interactions.. J Neurochem.

[21] Lernmark A (1996). Glutamic acid decarboxylase–gene to antigen to disease.. J Intern Med.

[22] Sha D, Jin Y, Wu H, Wei J, Lin CH (2008). Role of mu-calpain in proteolytic cleavage of brain L-glutamic acid decarboxylase.. Brain Res.

[23] Wei J, Lin CH, Wu H, Jin Y, Lee YH (2006). Activity-dependent cleavage of brain glutamic acid decarboxylase 65 by calpain.. J Neurochem.

[24] Monnerie H, Le Roux PD (2008). Glutamate alteration of glutamic acid decarboxylase (GAD) in GABAergic neurons: the role of cysteine proteases.. Exp Neurol.

[25] Monnerie H, Le Roux PD (2007). Reduced dendrite growth and altered glutamic acid decarboxylase (GAD) 65- and 67-kDa isoform protein expression from mouse cortical GABAergic neurons following excitotoxic injury in vitro.. Exp Neurol.

[26] Vanderklish PW, Bahr BA (2000). The pathogenic activation of calpain: a marker and mediator of cellular toxicity and disease states.. Int J Exp Pathol.

[27] Herrmann J, Lerman LO, Lerman A (2007). Ubiquitin and ubiquitin-like proteins in protein regulation.. Circ Res.

[28] Hershko A, Ciechanover A (1998). The ubiquitin system.. Annu Rev Biochem.

[29] Ge P, Luo Y, Liu CL, Hu B (2007). Protein aggregation and proteasome dysfunction after brain ischemia.. Stroke.

[30] Asai A, Tanahashi N, Qiu JH, Saito N, Chi S (2002). Selective proteasomal dysfunction in the hippocampal CA1 region after transient forebrain ischemia.. J Cereb Blood Flow Metab.

[31] Phillips JB, Williams AJ, Adams J, Elliott PJ, Tortella FC (2000). Proteasome inhibitor PS519 reduces infarction and attenuates leukocyte infiltration in a rat model of focal cerebral ischemia.. Stroke.

[32] Williams AJ, Hale SL, Moffett JR, Dave JR, Elliott PJ (2003). Delayed treatment with MLN519 reduces infarction and associated neurologic deficit caused by focal ischemic brain injury in rats via antiinflammatory mechanisms involving nuclear factor-kappaB activation, gliosis, and leukocyte infiltration.. J Cereb Blood Flow Metab.

[33] Zhang L, Zhang ZG, Zhang RL, Lu M, Adams J (2001). Postischemic (6-Hour) treatment with recombinant human tissue plasminogen activator and proteasome inhibitor PS-519 reduces infarction in a rat model of embolic focal cerebral ischemia.. Stroke.

[34] Solimena M, Dirkx R, Radzynski M, Mundigl O, De Camilli P (1994). A signal located within amino acids 1-27 of GAD65 is required for its targeting to the Golgi complex region.. J Cell Biol.

[35] Santos AR, Duarte CB (2008). Validation of internal control genes for expression studies: effects of the neurotrophin BDNF on hippocampal neurons.. J Neurosci Res.

[36] Kubista K, Sindelka R, Tichopad A, Bergkvist A, Lindh D (2007). The prime technique: real-time PCR data analysis, G.I.T.. Laboratory Journal.

[37] Bahr BA (2000). Integrin-type signaling has a distinct influence on NMDA-induced cytoskeletal disassembly.. J Neurosci Res.

[38] Munirathinam S, Rogers G, Bahr BA (2002). Positive modulation of alpha-amino-3-hydroxy-5-methyl-4-isoxazolepropionic acid-type glutamate receptors elicits neuroprotection after trimethyltin exposure in hippocampus.. Toxicol Appl Pharmacol.

[39] Frick KM, Burlingame LA, Delaney SS, Berger-Sweeney J (2002). Sex differences in neurochemical markers that correlate with behavior in aging mice.. Neurobiol Aging.

[40] Almeida RD, Manadas BJ, Melo CV, Gomes JR, Mendes CS (2005). Neuroprotection by BDNF against glutamate-induced apoptotic cell death is mediated by ERK and PI3-kinase pathways.. Cell Death Differ.

[41] Duarte CB, Ferreira IL, Santos PF, Oliveira CR, Carvalho AP (1993). Glutamate increases the [Ca^2+^]_i_ but stimulates Ca^2+^-independent release of [^3^H]GABA in cultured chick retina cells.. Brain Res.

[42] Swanwick CC, Harrison MB, Kapur J (2004). Synaptic and extrasynaptic localization of brain-derived neurotrophic factor and the tyrosine kinase B receptor in cultured hippocampal neurons.. J Comp Neurol.

[43] Kisselev AF, Goldberg AL (2001). Proteasome inhibitors: from research tools to drug candidates.. Chem Biol.

[44] Rock KL, Gramm C, Rothstein L, Clark K, Stein R (1994). Inhibitors of the proteasome block the degradation of most cell proteins and the generation of peptides presented on MHC class I molecules.. Cell.

[45] Myung J, Kim KB, Crews CM (2001). The ubiquitin-proteasome pathway and proteasome inhibitors.. Med Res Rev.

[46] Rideout HJ, Stefanis L (2002). Proteasomal inhibition-induced inclusion formation and death in cortical neurons require transcription and ubiquitination.. Mol Cell Neurosci.

[47] Butts BD, Hudson HR, Linseman DA, Le SS, Ryan KR (2005). Proteasome inhibition elicits a biphasic effect on neuronal apoptosis via differential regulation of pro-survival and pro-apoptotic transcription factors.. Mol Cell Neurosci.

[48] Myung J, Kim KB, Lindsten K, Dantuma NP, Crews CM (2001). Lack of proteasome active site allostery as revealed by subunit-specific inhibitors.. Mol Cell.

[49] Yang Y, Kitagaki J, Dai RM, Tsai YC, Lorick KL (2007). Inhibitors of ubiquitin-activating enzyme (E1), a new class of potential cancer therapeutics.. Cancer Res.

[50] Caba E, Brown QB, Kawasaki B, Bahr BA (2002). Peptidyl alpha-keto amide inhibitor of calpain blocks excitotoxic damage without affecting signal transduction events.. J Neurosci Res.

[51] Sorokin AV, Selyutina AA, Skabkin MA, Guryanov SG, Nazimov IV (2005). Proteasome-mediated cleavage of the Y-box-binding protein 1 is linked to DNA-damage stress response.. EMBO J.

[52] Baugh JM, Pilipenko EV (2004). 20S proteasome differentially alters translation of different mRNAs via the cleavage of eIF4F and eIF3.. Mol Cell.

[53] Martin SB, Waniewski RA, Battaglioli G, Martin DL (2003). Post-mortem degradation of brain glutamate decarboxylase, Neurochem Int.

[54] Hsu CC, Davis KM, Jin H, Foos T, Floor E (2000). Association of L-glutamic acid decarboxylase to the 70-kDa heat shock protein as a potential anchoring mechanism to synaptic vesicles, J Biol Chem.

[55] Esclapez M, Tillakaratne NJ, Kaufman DL, Tobin AJ, Houser CR (1994). Comparative localization of two forms of glutamic acid decarboxylase and their mRNAs in rat brain supports the concept of functional differences between the forms, J Neurosci.

[56] Battaglioli G, Liu H, Hauer CR, Martin DL (2005). Glutamate decarboxylase: loss of N-terminal segment does not affect homodimerization and determination of the oxidation state of cysteine residues, Neurochem Res 30,.

[57] Fenalti G, Law RH, Buckle AM, Langendorf C, Tuck K (2007). GABA production by glutamic acid decarboxylase is regulated by a dynamic catalytic loop.. Nat Struct Mol Biol.

[58] Salin P, Chesselet MF (1993). Expression of GAD (M_r_ 67,000) and its messenger RNA in basal ganglia and cerebral cortex after ischemic cortical lesions in rats.. Exp Neurol.

[59] Qin ZH, Zhang SP, Weiss B (1994). Dopaminergic and glutamatergic blocking drugs differentially regulate glutamic acid decarboxylase mRNA in mouse brain.. Brain Res Mol Brain Res.

[60] Jackman RW, Kandarian SC (2004). The molecular basis of skeletal muscle atrophy.. Am J Physiol Cell Physiol.

[61] Bartoli M, Richard I (2005). Calpains in muscle wasting.. Int J Biochem Cell Biol.

[62] Alkalay I, Yaron A, Hatzubai A, Orian A, Ciechanover A (1995). Stimulation-dependent I kappa B alpha phosphorylation marks the NF-kappa B inhibitor for degradation via the ubiquitin-proteasome pathway.. Proc Natl Acad Sci U S A.

[63] Scherer DC, Brockman JA, Chen Z, Maniatis T, Ballard DW (1995). Signal-induced degradation of I kappa B alpha requires site-specific ubiquitination.. Proc Natl Acad Sci U S A.

[64] Chen F, Lu Y, Kuhn DC, Maki M, Shi X (1997). Calpain contributes to silica-induced I kappa B-alpha degradation and nuclear factor-kappa B activation.. Arch Biochem Biophys.

[65] Di Napoli M, McLaughlin B (2005). The ubiquitin-proteasome system as a drug target in cerebrovascular disease: therapeutic potential of proteasome inhibitors.. Curr Opin Investig Drugs.

[66] Ray SK (2006). Currently evaluated calpain and caspase inhibitors for neuroprotection in experimental brain ischemia.. Curr Med Chem.

[67] Noga M, Hayashi T (1996). Ubiquitin gene expression following transient forebrain ischemia.. Brain Res Mol Brain Res.

[68] Vannucci SJ, Mummery R, Hawkes RB, Rider CC, Beesley PW (1998). Hypoxia-ischemia induces a rapid elevation of ubiquitin conjugate levels and ubiquitin immunoreactivity in the immature rat brain.. J Cereb Blood Flow Metab.

[69] Liu CL, Martone ME, Hu BR (2004). Protein ubiquitination in postsynaptic densities after transient cerebral ischemia.. J Cereb Blood Flow Metab.

[70] Shah IM, Lees KR, Pien CP, Elliott PJ (2002). Early clinical experience with the novel proteasome inhibitor PS-519.. Br J Clin Pharmacol.

[71] Wei J, Jin Y, Wu H, Sha D, Wu JY (2003). Identification and functional analysis of truncated human glutamic acid decarboxylase 65.. J Biomed Sci.

[72] Wei J, Wu JY (2008). Post-translational regulation of L-glutamic acid decarboxylase in the brain.. Neurochem Res.

[73] de Almeida OM, Gardino PF, Loureiro dos Santos NE, Yamasaki EN, de Mello MC (2002). Opposite roles of GABA and excitatory amino acids on the control of GAD expression in cultured retina cells, Brain Res.

[74] de Mello FG, Hokoc JN, Ventura AL, Gardino PF (1991). Glutamic acid decarboxylase of embryonic avian retina cells in culture: regulation by gamma-aminobutyric acid (GABA).. Cell Mol Neurobiol.

[75] Kanaani J, Ael-D el-Husseini, Aguilera-Moreno A, Diacovo JM, Bredt DS (2002). A combination of three distinct trafficking signals mediates axonal targeting and presynaptic clustering of GAD65.. J Cell Biol.

[76] Kanaani J, Patterson G, Schaufele F, Lippincott-Schwartz J, Baekkeskov S (2008). A palmitoylation cycle dynamically regulates partitioning of the GABA-synthesizing enzyme GAD65 between ER-Golgi and post-Golgi membranes, J Cell Sci.

[77] Soghomonian JJ, Martin DL (1998). Two isoforms of glutamate decarboxylase: why?. Trends Pharmacol Sci.

